# Motion of Molecular Probes and Viscosity Scaling in Polyelectrolyte Solutions at Physiological Ionic Strength

**DOI:** 10.1371/journal.pone.0161409

**Published:** 2016-08-18

**Authors:** Krzysztof Sozanski, Agnieszka Wisniewska, Tomasz Kalwarczyk, Anna Sznajder, Robert Holyst

**Affiliations:** Institute of Physical Chemistry, Polish Academy of Sciences, Kasprzaka 44/52, 01-224 Warsaw, Poland; Martin-Luther-Universitat Halle-Wittenberg, GERMANY

## Abstract

We investigate transport properties of model polyelectrolyte systems at physiological ionic strength (0.154 M). Covering a broad range of flow length scales—from diffusion of molecular probes to macroscopic viscous flow—we establish a single, continuous function describing the scale dependent viscosity of high-salt polyelectrolyte solutions. The data are consistent with the model developed previously for electrically neutral polymers in a good solvent. The presented approach merges the power-law scaling concepts of de Gennes with the idea of exponential length scale dependence of effective viscosity in complex liquids. The result is a simple and applicable description of transport properties of high-salt polyelectrolyte solutions at all length scales, valid for motion of single molecules as well as macroscopic flow of the complex liquid.

## Introduction

Over the last few decades, polymer science has been developing two parallel and apparently incompatible approaches to transport properties of polymer solutions. The first approach, centred on the bulk properties and macroscopic flow in such systems, stems from the scaling concepts introduced by de Gennes [1] and relies on power laws relating the solution viscosity to polymer concentration [2–6]. The second approach, focusing on probe motion in polymer systems, explores the length scale dependence of the effective viscosity experienced by the probe. There, the widely accepted model is a stretched exponential dependence of the effective viscosity on polymer concentration [7–10]. Recently, we proposed a model [11, 12] reconciling the two mathematically distinct approaches (algebraic and exponential), which allows for description of viscosity of polymer solutions across all length scales and different concentration regimes with a single, continuous function.

The model of length scale dependent viscosity [11, 12] is largely inspired by the early observations by Langevin and Rondelez [13] on probe sedimentation in polymer meshwork as well as Phillies’ suggestions on the ubiquity of stretched exponential dependencies in polymer systems [14–16]. According to the fundamental Stokes-Einstein relation, viscosity of a solution is inversely proportional to the diffusion coefficient of a probe freely diffusing in this solution. However, in complex liquids apparent enhancement of diffusion of relatively small probes is universally observed [11, 17–22]. This effect is crucial for accurate description of diffusion rates in living cells [23–25]. Deviations from the Stokes-Einstein relation become significant when the hydrodynamic radius of the probe *r*_p_ drops below the characteristic length scale of the liquid—in polymer systems, comparable to the gyration radius of a polymer coil *R*_g_ [11, 21, 26]. Theoretical considerations of mobility of nanoprobes in polymer liquids predict qualitative shifts in the probe’s behaviour upon crossing between different length-scale regimes [27]. The crucial length scale featured in polymer solution is identified as correlation length, *ξ*, also interpreted as the blob size in entangled systems [1, 5, 28]. It is calculated as

$$\xi =R_{g}{\left(\frac{c}{c^{*}}\right)}^{-\beta }$$ (1)

where *β* is a scaling exponent equal to 0.75 for flexible chains in a good solvent. c* denotes the overlap concentration, usually defined as [1, 5]

$$c^{*}=\frac{M_{w}}{4/3\pi R_{g}^{3}N_{A}}$$ (2)

According to Cai et al. [27], small probes (size *d* below *ξ*) should diffuse in a polymer solution similarly as in pure solvent, while large probes (*d* greater than the tube diameter) should experience bulk viscosity of the solution. In the intermediate size range, an effective viscosity value is estimated as proportional to (*d*/*ξ*)^2^.

In our previous work [11, 12] we have demonstrated experimentally that indeed the viscosity experienced by the probe changes with the probe size, with solvent viscosity and bulk solution viscosity being the two limiting values. However, there is a smooth, gradual dependence of the effective viscosity on the probe size rather than abrupt changes upon crossing certain length scale markers. A universal, semi-empirical formula has been proposed to describe viscosity, *η*, across all length scales [11, 12]:

$$\eta =\eta_{0}\exp\left[b{\left(\frac{R_{\text{eff}}}{\xi }\right)}^{a}\right]$$ (3)

where *η*_0_—solvent viscosity, *a*, *b*—parameters of the order of unity. *R*_eff_ is the effective probe radius [12, 25], dependent on the hydrodynamic radius of the polymer *R*_h_ and actual probe size *r*_p_ as $R_{\text{eff}}^{-2}=R_{h}^{-2}+r_{p}^{-2}$. In the large probe limit, *R*_eff_ → *R*_h_ and the equation describes the macroscopic viscosity of the solution.

The general dependence given by Eq 3 was developed for electrically neutral polymer solutions. Its applicability was also extended to other crowded systems, such as colloidal solutions and cellular cytoplasm [25, 29]. However, until now, it has not been directly applied to polyelectrolyte systems, which constitute an important branch of modern polymer science.

The recent revival of interest in dynamics of polyelectrolyte solutions and motion in charged systems is not only due to purely physical appeal of these issues, but also their biological context [30–33]. Most of the filamentous biopolymers such as DNA, RNA, F-actin, or microtubules have a net negative charge distributed along the chain [33]. In fact, nucleic acids with a linear charge density of about 6 *e*/nm are among the most highly charged linear polyelectrolytes known [34]. Also, the distribution of isoelectric points of proteins is usually bimodal with a minimum around neutral pH [35], indicating that most proteins are charged at physiological conditions. Electrostatic interactions, due to their relatively long range, high energy, and ubiquity may have a crucial impact on transport properties of biological complex liquids. In fact, it has been shown that even in a simple case of solutions of hard spheres the charge of the colloidal particles has a significant impact on the viscosity of the solution [29, 36]. However, recent studies of mobility in crowded environment tend to focus on the mesoscopic structure of liquids and length scale dependencies, disregarding the electrostatics. Nevertheless, there are no systematic studies available that would clearly validate such simplification.

A convenient model system for studying crowded, charged systems is a polyelectrolyte solution. Because of repulsion between like charges on the monomers, polyelectrolytes dissolved in polar solvents tend to acquire a more stretched, rod-like conformation than neutral polymers—especially at low ionic strength of the solution (in the low salt regime). This entails significant consequences for behaviour of the chains, including relatively low overlap concentrations and high entanglement concentrations of polyelectrolytes [37], peculiar dependence of reduced viscosity on concentration [38], charge density influence on the chain conformation [39, 40], or counter-ion condensation effects [41]. Detailed insights into the structure and dynamics of polyelectrolyte solutions can be found e.g. in the extensive reviews by Dobrynin and Rubinstein [37] as well as Colby [42].

There is a large body of work—both theoretical and experimental—on viscosity of polyelectrolyte solutions [43–51]. Various scaling approaches to the viscosity-concentration dependence have been developed, often related to the Fuoss power law (*η* ∝ *c*^1/2^). However, most studies concern salt-free conditions, where strong intra- and inter-chain repulsion is observed. This leads to an extremely broad concentration regime (up to three orders of magnitude) [37, 51] where the chains are overlapped, but not entangled. It has been suggested that some anisotropic, layer-like structures may be formed in such systems [51–53], which poses additional difficulties for universal description of transport properties of such systems. Moreover, if no measures are taken to remove carbon dioxide, the solutions are not actually salt-free [44], which may be a source of experimental errors. At very low ionic strength, polyelectrolyte systems are extremely fragile to even slight amounts of ions. Although appealing and challenging from the theoretical point of view, for most industrially and biologically relevant problems the salt-free case is of only limited interest. Yet, relatively little attention has until now been given to the high salt limit conditions (high ionic strength). Obviously, with increasing ionic strength the Debye length decreases and charge screening gains a heavy influence on the polyelectrolyte chain properties. Polyelectrolyte solutions at the high salt limit should therefore belong to the same universality class as non-charged polymers. However, it is not obvious whether their transport properties can be described identically as those of neutral polymer systems.

There is a number of studies of probe diffusion in polyelectrolyte solutions [17, 54–58]. Qualitatively similar observations concerning apparent breach of the Stokes-Einstein relation are made as in case of non-charged systems. The presence of charges in the system influences the probe diffusion in two distinct ways. One is related to the structure of the polymer mesh itself, via backbone stiffening and inter-chain repulsion. The other is related to the probe-polymer interactions, which—especially in case of electrostatic attraction between the probe and mesh—may vastly influence the probe mobility even at very low polyelectrolyte concentrations [57].

Herein, we apply the paradigm of length scale dependent viscosity [12] to describe the transport properties of polyelectrolyte solutions. Ultimately aiming to develop an accurate and applicable model for description of molecular mobility in living cells, we restrict ourselves to the physiological ionic strength conditions (0.154 M). This lies within the high salt regime and entails substantial charge screening. The crucial question posed here is whether polyelectrolyte chains at such conditions can be treated as flexible, non-charged chains, quantitatively subject to the same viscosity scaling relations.

We present results of measurements of viscosity of a range of high-salt polyelectrolyte solutions (performed using rotational rheometry) as well as data on probe diffusion in such systems (obtained using fluorescence correlation spectroscopy, FCS). Irrespective of the flow length scale (from macroscopic viscous flow to diffusion of nm-sized probes), all the data are described within a single, continuous scaling equation. To avoid possible artefacts related to adsorption of probes on the polyelectrolyte mesh, in the FCS studies we only use probes that at pH of 7.4 are either electrically neutral or charged negatively (i.e. have the same charge sign as the crowding polyelectrolyte). We postulate that the viscosity scaling approach developed for non-charged systems (Eq 3) is perfectly applicable to polyelectrolytes at high salt conditions, with the entanglement-related crossover clearly displayed. According to our previous observations [59], the crossover point between these two regimes lies at the polymer concentration such that *ξ* = *R*_h_. This falls above the overlap concentration c* and seems to be the crucial shifting point for description of transport properties of polymer solutions. Interestingly, it was found for non-charged systems that all the solutions of lower concentration are described within a single scaling curve, with no change of equation form or parameters around c* [59].

The scaling exponents *a* for the two concentration regimes—below and above the *ξ* = *R*_h_ point—are in case of polyelectrolyte solutions the same as for non-charged polymers. Only slight variations in the fitted, interaction-dependent parameter *b* are observed. We use the macroscopic viscosity data to test the theoretical model based on the electrostatic blob scaling theory [47, 60, 61]. The semi-empirical approach we propose provides an accurate and applicable description of transport properties of real high-salt polyelectrolyte solutions across all length scales.

## Materials and Methods

Two model polyelectrolytes were investigated: poly(methacrylic acid) sodium salt, PMAANa, and poly(styrene sulfonate) sodium salt, PSSNa. Polymers were purchased as molecular weight standards from Polymer Standards Systems, Mainz, Germany. Sample designations refer to approximate molecular mass of the polymers, which were 7, 19, 35, 143, and 311 kDa for PMAANa and 61, 322, and 666 kDa for PSSNa (i.e. linear chains of up to about 3000 monomers in both cases). Exact data on the average molecular masses of the polymers is available in S1 Table. In aqueous solutions both these polyelectrolytes dissociate, leaving negative charges on the backbone (COO^−^ groups). Concentrations of the investigated solutions were selected to fit within the dilute and semi-dilute regime [59], i.e. below the onset of melt-like concentrated regime (6-10 times the overlap concentration [5, 62]).

10 mM phosphate buffer (pH 7.4) of ionic strength adjusted with NaCl to 0.154 M (PBS buffer) was used as solvent in all experiments. PBS is frequently utilized to mimic physiological conditions. The Debye screening length for such solution is 0.78 nm. Samples were prepared at room temperature and stirred for at least an hour to ensure full dissolution of the polymers and equilibration of the solutions.

Macroscopic viscosity was measured using a Malvern Kinexus Pro rotational rheometer, with a cone-plate geometry. For each sample, a shear stress vs. shear rate dependence was first measured to establish the range of fully Newtonian response of the sample. In this range, viscosity measurements were performed at controlled shear stress and linear extrapolation to viscosity at zero shear stress was performed. Temperature was kept at 25 ± 0.1°C.

Fluorescence correlation spectroscopy (FCS) measurements were performed using a system based on a Nikon C1 confocal laser scanning microscope, equipped with a Nikon PlanApo 60x objective (NA = 1.2) and a PicoQuant LSM upgrade system (featuring two SPAD detectors working in parallel, PicoHarp 300 TCSPC module and SymPhoTime software). Temperature was kept at 25 ± 0.1°C within a box enclosure (OkoLab). Depending on the probe, 488 nm diode laser (PicoQuant) or 543 nm He-Ne laser (Melles Griot) was used as excitation light source. For each experiment, at least 10 autocorrelation curves (30-60 s acquisition time each) were recorded; cumulated fitting was performed in Gnuplot software. Single-component model assuming Gaussian detection volume and including triplet states was used for data fitting [63].

A range of fluorescent probes was used for the FCS experiments; they are listed in Table 1 along with their hydrodynamic radii and charges. Rhodamine dyes as well as apoferritin were purchased form Sigma. Protein labelling was performed using Atto 488 NHS-ester (Atto-Tec GmbH) according to the protocol suggested by the manufacturer. Labelled protein was purified via size-exclusion chromatography (Bio-Gel P-30, Bio-Rad). Dextrans (of average molecular mass 4.4 and 155 kDa) labelled with tetramethylrhodamine were purchased from Sigma and used as obtained.

**Table 1:** Hydrodynamic radii of the probes used throughout the FCS experiments (*r*_p_), along with the probe charges at the pH of phosphate buffer (7.4).

Probe	*r*_p_ [nm]	Charge [*e*]
Rhodamine 110 (Rho110)	0.52	0^a^
Rhodamine B (RhoB)	0.58	0^a^
Dextran 4.4 kDa	1.3	0^b^
Apoferritin	6.9	negative^c^
Dextran 155 kDa	7.3	0^b^

^*a*^ Zwitterionic; effective charge close to 0 at neutral pH

^*b*^ Neutral polymer labelled with TAMRA (zwitterionic dye; effective charge close to 0 at neutral pH)

^*c*^ Isoelectric point of the protein around 5; each Atto 488 label adds a -1 charge; on average 1–2 labels per molecule

## Results and Discussion

The starting point for empirical construction of scaling relations is determination of the basic parameters of the polymer systems. To establish the length scales of the polyelectrolyte systems, we first performed accurate measurements of shear viscosity of low-concentration polyelectrolyte solutions in PBS (several points below the expected overlap concentration for each polyelectrolyte). Reduced viscosities were calculated by dividing the solution viscosity by the buffer viscosity measured in the same conditions. Then, for each polyelectrolyte the reduced viscosities diminished by 1 were plotted as a function of the polyelectrolyte concentration. A straight line was fitted (with no intercept allowed), whose slope was taken as the intrinsic viscosity [*η*] value for a given polyelectrolyte. Overlap concentration was calculated as [*η*]^−1^ [5]. On this basis, gyration radii of the polymer coils *R*_g_ were estimated as [5, 64]

$$R_{g}={\left(\frac{3M_{w}}{4\pi c^{*}}\right)}^{1/3}$$ (4)

Hydrodynamic radii of the polymer coils *R*_h_ were estimated according to the Kirkwood-Riseman theory [65] as 0.665*R*_g_. The obtained values of intrinsic viscosity, overlap concentration, as well as dimensions of polymer chains are available in the S1 Table. All the aforementioned calculations presume that polyelectrolyte chain in aqueous solution at high salt concentration behaves similarly to a neutral chain in a good solvent. Experimental validation of such assumption lies in good conformity of the results presented hereby with the scaling model developed for flexible polymers in a good solvent.

Further viscosity measurements were extended to span both the non-entangled and entangled concentration regimes, i.e. below and above the point where the correlation length *ξ* equals the hydrodynamic radius of the coil *R*_h_ [59]. As was shown for neutral polymer in a good solvent (aqueous solutions of polyethylene glycol, PEG), Eq 3 can be used in both these regimes, with scaling exponent *a* changing from 1.29 in non-entangled systems to 0.78 in entangled ones [59]. *R*_eff_ reduces itself in case of macroscopic flow to *R*_h_. Parameter *b* was established as equal to 1.61 for macroscale flow of PEG aqueous solutions. It was suggested that *a* depends on the structure and topology of the complex liquid, while *b* is related to the activation energy for the viscous flow of the liquid [66] and is therefore dependent on the strength of the polymer–polymer and polymer–solvent interactions.

**Fig 1:**
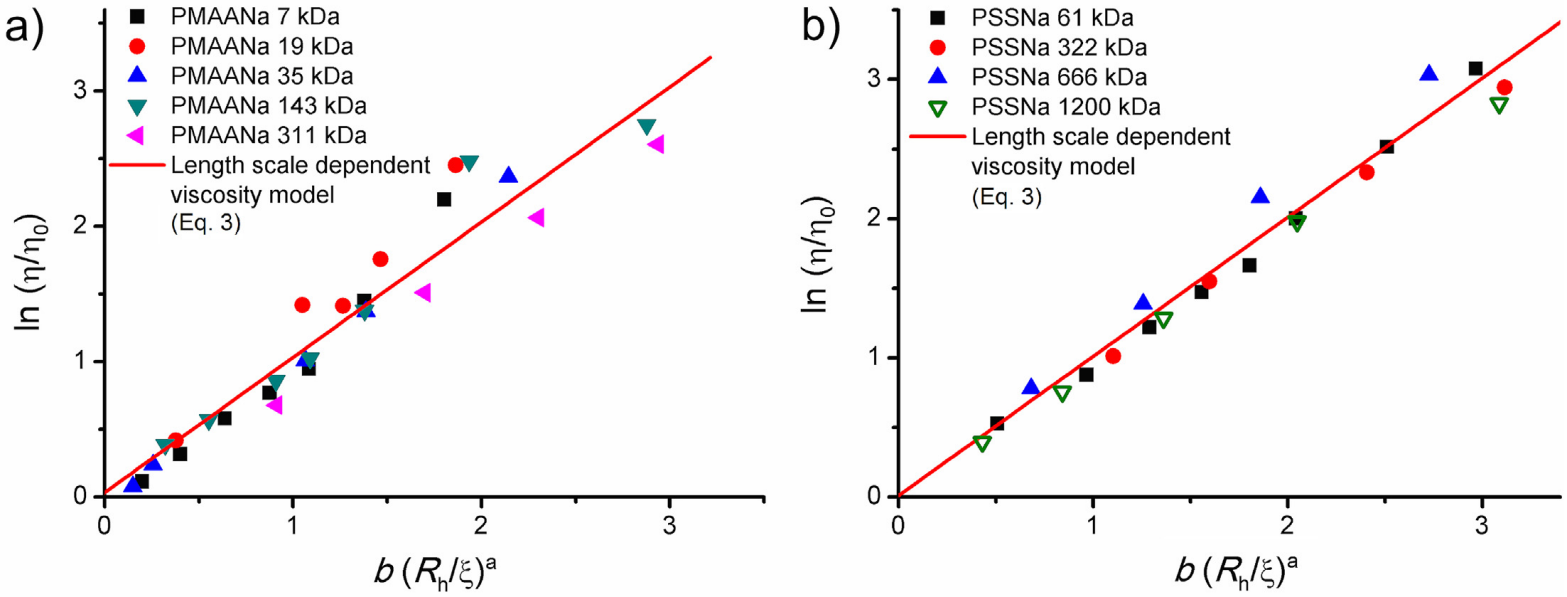
Macroviscosity measurements. Results of measurements of macroscopic viscosity (rotational rheometry) of aqueous solutions of a) PMAANa and b) PSSNa at ionic strength of 0.154 M and pH of 7.4. Good conformity with the model originally developed for neutral polymer solutions (Eq 3, solid line) is observed in both cases. In panel b) literature data from [61] are included (empty symbols). These data correspond to viscosity measurements on a 1200 kDa PSSNa sample at 0.01 M NaCl. This still falls within the high salt regime and the results follow the model proposed hereby.

We applied Eq 3 to the results of macroscopic viscosity measurements of PMAANa and PSSNa solutions. The values of *a* for the non-entangled and entangled regimes were kept the same as for the PEG systems (consistently keeping the high-salt approximation of non-charged, flexible chains). *b* was treated as a fitting parameter for both polymers. As can be seen in Fig 1, the model describes the data well across the whole studied concentration range. The fitted values of *b* equal to 1.09 and 1.02 for PMAANa and PSSNa, respectively.

The value of structure-related exponent *a* obtained for flexible chains (PEG) readily describes the presented data. It is a strong indication that, from the point of view of macroscopic flow, the PMAANa and PSSNa solutions at physiological ionic strength behave as if there were no charges along the polymer backbone: the charge screening is indeed sufficient to elasticize the chains. This stands to reason, since the correlation length was in all the investigated systems significantly greater than the range of screening of electrostatic interactions (typically, *ξ* of several to tens of nm compared to the Debye length of 0.78 nm). Therefore, the hydrodynamic interactions prevail over the electrostatics. Also, the interaction-dependent *b* parameter fitted for both the polyelectrolytes is of the same order of magnitude as for PEG (cf. S2 Table).

We matched our data on bulk viscosity of the polyelectrolyte solutions against Dobrynin’s simple power law [47], based on de Gennes’ concept of scaling of electrostatic blobs [60]. According to this approach, for non-entangled, semidilute solutions of high-salt polyelectrolytes, viscosity should be proportional to $c^{5/4}c_{s}^{-3/4}N$, where *c* is the molar monomer concentration, *c*_*s*_ is the molar concentration of the added salt and *N* is the number of monomers in a chain. Putting *α* as an independent proportionality factor allowing to write an equation, we obtain

$$\eta =\alpha c^{5/4}c_{s}^{-3/4}N$$ (5)

**Fig 2:**
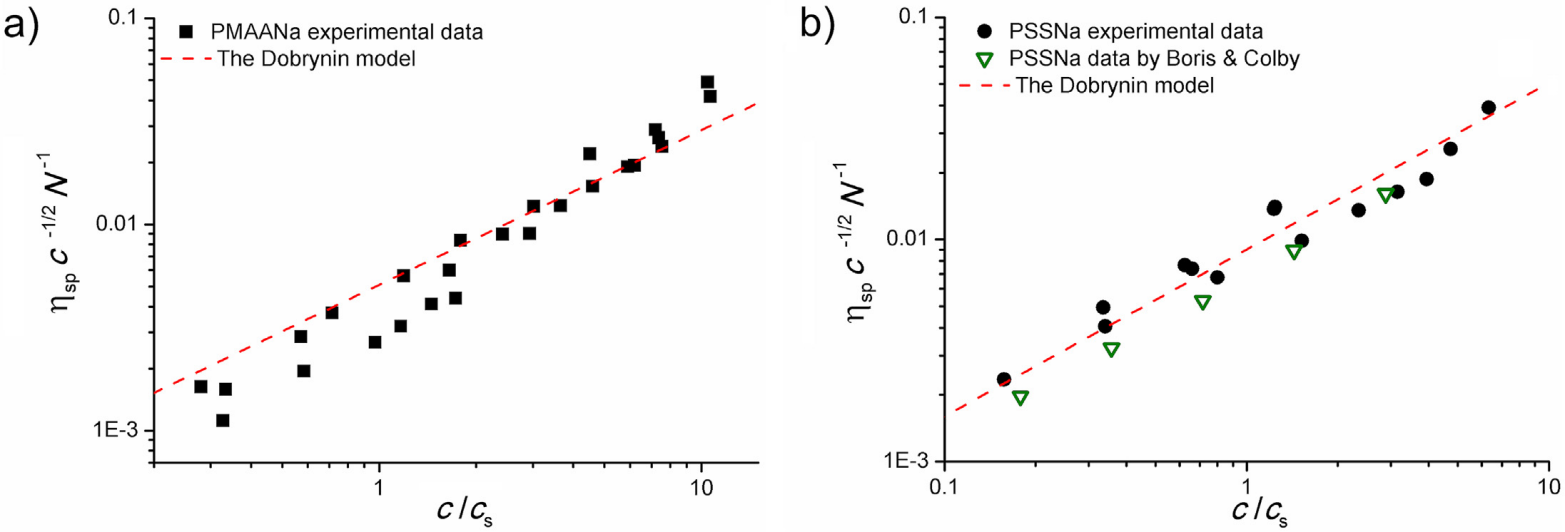
Comparison with the theoretical model. Bulk viscosity data for all the investigated solutions of a) PMAANa and b) PSSNa plotted according to Dobrynin’s theoretical model [47] based on de Gennes’ concept of scaling of electrostatic blobs [60]—Eq 5. Panel b) includes also data from [61]. Despite some deviations, the model seems to describe the data acceptably well.

Following the approach of Boris and Colby [61], we plotted the specific viscosity *η*_*sp*_ divided by *c*^−1/2^ *N* as a function of *c*/*c*_*s*_ on a log-log plot. Since for this analysis we intended to reproduce the scaling proposed in [61], we treated all our datapoints as if they belonged within the non-entangled regime, along the lines of the entanglement definition used therein. The result is shown in Fig 2. In each plot, results obtained for all the molecular weights of a given polymer are cumulated. Additionally, we include the high-salt viscosity data from [61]. According to the theory [61], the points in each plot should fall along a straight line of a slope of 3/4. Both in the case of PMAANa and PSSNa a general dependence of the implied kind is observed. However, there is a systematic deviation from the straight, marked more clearly for PMAANa. Moreover, coefficient *α* read as the intercept of the straight (fitted with slope fixed at 3/4) is significantly different for the two polymers, amounting to 0.05 and 0.009 for PMAANa and PSSNa, respectively. This suggests that quantitative description of real polyelectrolyte solutions still requires system-dependent optimization of the scaling parameters.

Thus, both the Dobrynin power law (Eq 5) and the model proposed hereby (Eq 3) feature one fitted, system-dependent parameter and a fixed exponent value. Also, both provide a reasonable description of the experimental data on bulk viscosity of polyelectrolyte solutions. However, applicability of Eq 5 is by default limited to such data. On the contrary, bulk liquid viscosity is only one of the limiting cases of Eq 3. The main feature of the proposed model is its straightforward applicability to motion of molecular probes. To validate it, we performed FCS experiments on the high-salt polyelectrolyte solutions, observing diffusion of several fluorescent probes (see Table 1) that revealed no tendency to attach to the polyelectrolyte mesh. Representative series of raw autocorrelation curves along with the fitted model are given in S1 Fig. To enable direct application of the viscosity scaling paradigm to the measured diffusion coefficients *D*, we chose to analyse and present the data in terms of effective viscosity (experienced by the probe). To do so, we used the simple dependence

$$\frac{\eta }{\eta_{0}}=\frac{D_{0}}{D}$$ (6)

where values with the 0 index refer to pure solvent. Eq 6 stems from the Stokes-Einstein relation, which can be applied to diffusion in complex liquids on condition that *η* is treated as a function of the probe size [11].

**Fig 3:**
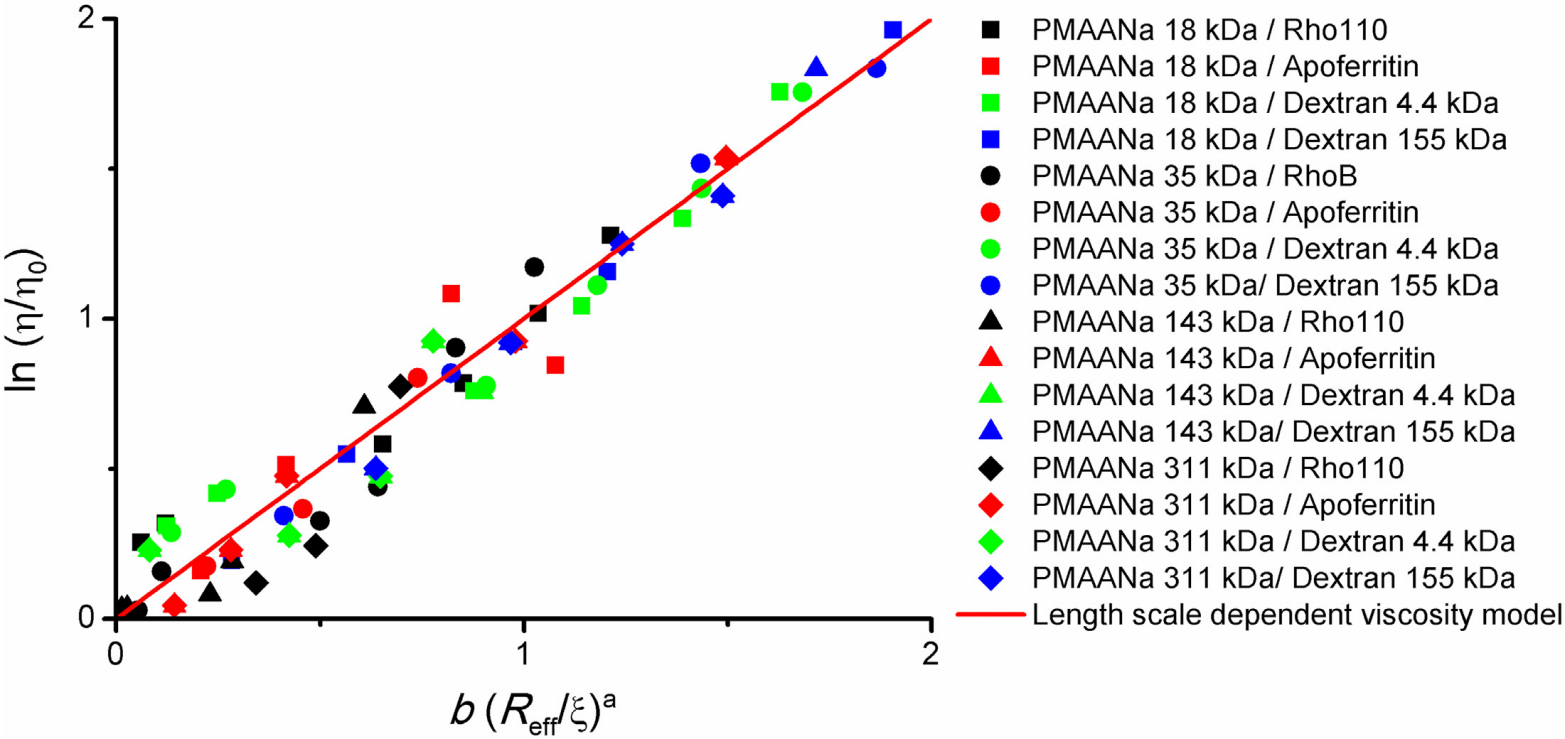
Nanoviscosity measurements. Results of fluorescence correlation spectroscopy (FCS) measurements of probe diffusion rates in solutions of PMAANa of different molecular masses. The probes used were rhodamine dyes, apoferritin and TAMRA-labelled dextrans. Ionic strength was kept at 0.154 M. Diffusion coefficients were translated to effective viscosities experienced by the probes via Eq 6. The data are plotted according to the model from Eq 3. All the probes are of neutral or negative electric charge (no electrostatic attraction to the polyelectrolyte chains).

Same as in case of the macroscopic viscosity, we fixed the *a* values to 1.29 and 0.78 for non-entangled and entangled systems, respectively, and left *b* as the only fitted parameter for each polymer–probe system. The results obtained for diffusion of negatively charged and electrically neutral probes in PMAANa solutions, plotted according to Eq 3, are given in Fig 3. Again, satisfactory conformity between the results and the model is readily observed. This indicates that if there is no electrostatic attraction between the probe and the polyelectrolyte chain, Eq 3 can be applied to accurately describe probe diffusion in polyelectrolyte systems in the high salt regime.

The values of *b* fitted individually for each polymer–probe pair range from 0.7 to 4.3 (all the fitted *b* values are available the S2 Table). These values are similar to what was observed for diffusion in PEG solutions [66]. Also, as already noted previously [66], a general increase of *b* values with decreasing *r*_p_/*R*_h_ ratio can be observed. However, since *b* is an interaction-dependent parameter [29], sheer length scale analysis does not explain its changes in full—further studies are necessary to quantitatively describe the variability of this parameter.

Let us consider the limiting cases of Eq 3 applied to motion in polyelectrolyte solutions. For large probes (*r*_p_ ≫ *R*_h_), effective radius *R*_eff_ becomes congruent with the polymer hydrodynamic radius *R*_h_ and macroscopic viscosity is observed. In this case, the relative viscosity of the solution is given by a stretched exponential function:

$$\frac{\eta }{\eta_{0}}=\exp\left[b{\left(\frac{R_{h}}{\xi }\right)}^{a}\right]$$ (7)

Importantly, for low polyelectrolyte concentrations (*c* ≪ c*), the *R*_h_/*ξ* ratio is small and the above equation can be approximated by the first terms of the series expansion to yield

$$\frac{\eta }{\eta_{0}}≃1+b{\left(\frac{R_{h}}{\xi }\right)}^{a}$$ (8)

where *a* = 1.29. Thus, we reach an algebraic form for the solution viscosity.

In the limit of small probe (*r*_p_ ≪ *R*_h_), effective radius is congruent with the probe radius and falls well below the characteristic length scales of the solution (*R*_eff_ = *r*_p_ ≪ *R*_h_, *ξ*). Then, the series approximation is justified, yielding again an algebraic dependence:

$$\frac{\eta }{\eta_{0}}≃1+b{\left(\frac{r_{p}}{\xi }\right)}^{a}$$ (9)

where *a* shifts from 0.78 in entangled solutions (*ξ* < *R*_h_) to 1.29 in non-entangled solutions (*ξ* > *R*_h_). Ultimately, when *r*_p_ → 0, the viscosity experienced by the probe is equal to the solvent viscosity, i.e. the motion of the probe is unaffected by the presence of the polyelectrolyte. This of course is valid as long as the solvent is treated as a continuous medium. Therefore, the extreme case of probe similar in size or smaller than the solvent molecule should be excluded from this reasoning—some anomalous effects may occur in such cases (such as e.g. the Grotthuss mechanism of proton diffusion in water).

In this study, no FCS results are reported for PSSNa solutions. This is due to residual autofluorescence of these solutions, which caused a significant increase of the background during the measurements. In regular UV-Vis spectroscopic measurements, both PSS and PMAA reveal absorption around 200–250 nm (manufacturer data and [67]), which is far from the 488 nm excitation wavelength used in this study. However, fluorescence in FCS is recorded with extremely high sensitivity—detection is realized at a single photon level. Since in the performed experiments the concentration (by weight) of probes is lower than the polymer concentration by 6-8 orders of magnitude, even a slight amount of dimly fluorescent background from the polymer precludes quantitative measurements. In this case, the background autofluorescence probably stems from either a low-intensity tail of the polymers’ excitation spectrum (not possible to resolve in regular UV-Vis) or post-synthetic impurities in the polyelectrolyte. We did not manage to fully remove the impurities by means of extensive dialysis nor find any wavelength in the visible spectrum where the background autofluorescence issue would be negligible.

For the same reason no probe diffusion results are reported for the 7 kDa PMAANa solutions: for such short polymer, the concentrations necessary to cause significant increase in viscosity had to be high (up to 30% by weight). Moreover, small chains are difficult to clean from trace impurities, similar in size. Therefore, also in this case we faced background autofluorescence levels that could significantly influence the results.

## Conclusions

On the basis of measurements of diffusion rates of molecular probes as well as shear viscosity, we validate application of Eq 3—a continuous equation describing transport properties of complex systems across all flow length scales—to high-salt polyelectrolyte solutions. Our model reconciles the two approaches frequently found in the literature, namely the algebraic and exponential scaling of effective viscosity with polymer concentration. The proposed relation was originally developed for complex liquids with electrically neutral crowders. Here, we experimentally show that it can be readily applied to high-salt polyelectrolyte solutions. We compare it with the theoretical predictions concerning bulk viscosity of the solutions, based on the simple electrostatic blob scaling—Eq 5. We find our data to qualitatively match that model. However, applicability of Eq 5 is intrinsically limited to the bulk viscosity of polyelectrolyte solutions. In case of the model suggested hereby (Eq 3), this is only one of the limiting cases. Eq 3 readily provides an accurate description of transport properties of complex liquids across all length scales: from macroscopic flow to diffusion of molecular probes. Moreover, applicability of Eq 3 is not limited to polymer science—it has already been shown to be relevant to micellar and hard sphere solutions, protein solutions, and even cellular cytoplasm. In this contribution, we scrutinize its validity in simple polyelectrolyte systems at physiological ionic strength, opening a new way towards quantitative description of motion of charged particles in biological systems.

The scaling exponent *a* appearing in Eq 3 previously established experimentally for PEG solutions still holds for the investigated high-salt polyelectrolyte systems. Since the general interpretation of this exponent is related to the mesoscopic structure and stiffness of the macromolecular system, it indicates that indeed the screened charges do not influence the overall physical properties of the system significantly. This observation validates the application of the simple formalism developed for description of physical properties of neutral polymer solutions to a whole new range of systems: high-salt regime of flexible polyelectrolyte solutions. Importantly, both in scientific and industrial applications of polyelectrolytes (biological and biomimetic systems, cosmetics, food industry, etc.) it is often the case that the high-salt condition is satisfied. On the other hand, the frequently investigated in theoretical works no-salt regime is extremely difficult to maintain in experimental conditions due to absorption of carbon dioxide from air or trace ionic impurities from sample containers.

We hereby provide a single, continuous function describing flow in high-salt polyelectrolyte solutions over all length scales. However, in case of observation of molecular motion, careful choice of probes is crucial: the fact that charge screening is sufficient to elasticize the polyelectrolyte chains does not preclude attractive interaction between probe and crowder of opposite charges. In fact, comparison of the diffusion rates predicted by the model given hereby with diffusion coefficients measured for probes interacting with the crowders (of opposite charges) should allow for quantitative elucidation of the equilibrium and rate constants of probe attachment to the crowders [68, 69]. This opens a way towards systematic studies of the influence of electrostatic attraction on (macro)molecular mobility and reactivity in crowded, biologically relevant systems. Further studies on models as well as experimental systems allowing for such in-depth analysis are currently in progress.

## Supporting Information

S1 Table Data on molecular mass standard grade polymers used in this study. (PDF)

S2 Table Scaling parameters obtained for the different studied systems. (PDF)

S1 Fig Representative autocorrelation data form fluorescence correlation spectroscopy measurements along with fitted curves. (PDF)
