# Supplementary material for: Motion of Molecular Probes and Viscosity Scaling in Polyelectrolyte Solutions at Physiological Ionic Strength
Source: PLoS One. 2016 Aug 18;11(8):e0161409. doi: 10.1371/journal.pone.0161409 (PMC4990340; doi:10.1371/journal.pone.0161409)
Supplement: S1 Table — (PDF) [file pone.0161409.s001.pdf]

S1 Table – Polymer characterization

Table S1. Data on molecular mass standard grade polymers used in this study: poly(methacrylic acid) sodium salt, PMAANa and poly(styrene sulfonate) sodium salt, PSSNa. M_w – weight-average molecular mass, as reported by the manufacturer; c^* – overlap concentration; R_g – gyration radius of a polymer coil in solution; R_h – hydrodynamic radius

Designation	M_w [Da]	c^* [g/cm^3]	R_g [nm]	R_h [nm]
**PMAANa**				
7 kDa	6660	0.084	3.15	2.09
19 kDa	18600	0.049	5.31	3.53
35 kDa	34700	0.025	8.18	5.44
143 kDa	143000	0.012	17.0	11.3
311 kDa	311000	0.0033	33.4	22.2
**PSSNa**				
61 kDa	60600	0.031	9.19	6.11
322 kDa	321600	0.0054	28.7	19.1
666 kDa	666000	0.0044	39.2	26.1

Polymers were purchased as molecular mass standards from Polymer Standards Systems, Mainz, Germany. Sample designations refer to approximate molecular masses of the polymers, which are 6.5, 18, 35, 140, and 310 kDa for PMAANa and 60, 320, and 670 kDa for PSSNa (i.e. linear chains of up to about 3000 monomers in both cases). According to the manufacturer's information, polydispersity indices for all the investigated polyelectrolytes are below 1.20. Assessment of molecular weights of PMAANa and PSSNa is based on gel permeation chromatography performed on parent poly(t-butyl methacrylate) and polystyrene, respectively.

Values of overlap concentration c^* as well as gyration and hydrodynamic radii of the given polyelectrolytes were inferred from accurate measurements of shear viscosity of their solutions at low concentrations (calculation procedure is described in the main text).
