# Supplementary material for: Motion of Molecular Probes and Viscosity Scaling in Polyelectrolyte Solutions at Physiological Ionic Strength
Source: PLoS One. 2016 Aug 18;11(8):e0161409. doi: 10.1371/journal.pone.0161409 (PMC4990340; doi:10.1371/journal.pone.0161409)
Supplement: S2 Table — (PDF) [file pone.0161409.s002.pdf]

S2 Table – Scaling parameters

Table S2. Values of the dimensionless parameter b (Eq 3, main text) fitted for the different studied systems

Polymer \ Probe	Rhodamine	Apoferritin	Dextran 4.4 kDa	Dextran 155 kDa	Macroscopic flow
**PMAANa**					
7 kDa	–	–	–	–	1.09
19 kDa	2.9	0.77	2.05	1.29	
35 kDa	2.13	0.66	1.95	1.13	
143 kDa	2.46	1.26	1.85	1.01	
311 kDa	4.34	3.77	2.04	0.79	
**PSSNa**					
61 kDa	–	–	–	–	1.02
322 kDa	–	–	–	–	
666 kDa	–	–	–	–	

All the data on macro- as well as nanoscopic viscosity was confronted with the scaling equation originally developed for non-charged systems (Eq 3, main text). There are two parameters in the formula, a and b. Exponent a is related to the mesoscopic structure and stiffness of the macromolecular system. We directly used fixed values of a as established previously for poly(ethylene glycol) solutions, i.e. 1.29 for non-entangled systems and 0.78 for entangled systems. We obtained very good linearity of the scaling plots, which indicates that indeed the investigated polyelectrolyte chains behave at physiological salt concentration as flexible, non-charged chains. Parameter b is suspected to depend on the polymer-polymer and polymer-solvent interactions in the case of macroscopic flow. For probe diffusion, it also accounts for the probe-crowder interactions and seems to be related to the size ratio of the probe and the crowder ($r_\mathrm{p}/R_\mathrm{h}$). However, at this time we are not able to provide an analytical formula describing it. We fitted the values of b to the results of the viscosity and diffusion coefficient measurements, independently for each of the systems. In every case, b was the only fitted parameter in the equation. All the obtained values are of the order of unity. They are listed in Table S2.
