# Supplementary material for: Motion of Molecular Probes and Viscosity Scaling in Polyelectrolyte Solutions at Physiological Ionic Strength
Source: PLoS One. 2016 Aug 18;11(8):e0161409. doi: 10.1371/journal.pone.0161409 (PMC4990340; doi:10.1371/journal.pone.0161409)
Supplement: S1 Fig — (PDF) [file pone.0161409.s003.pdf]

Figure S1 – FCS curves

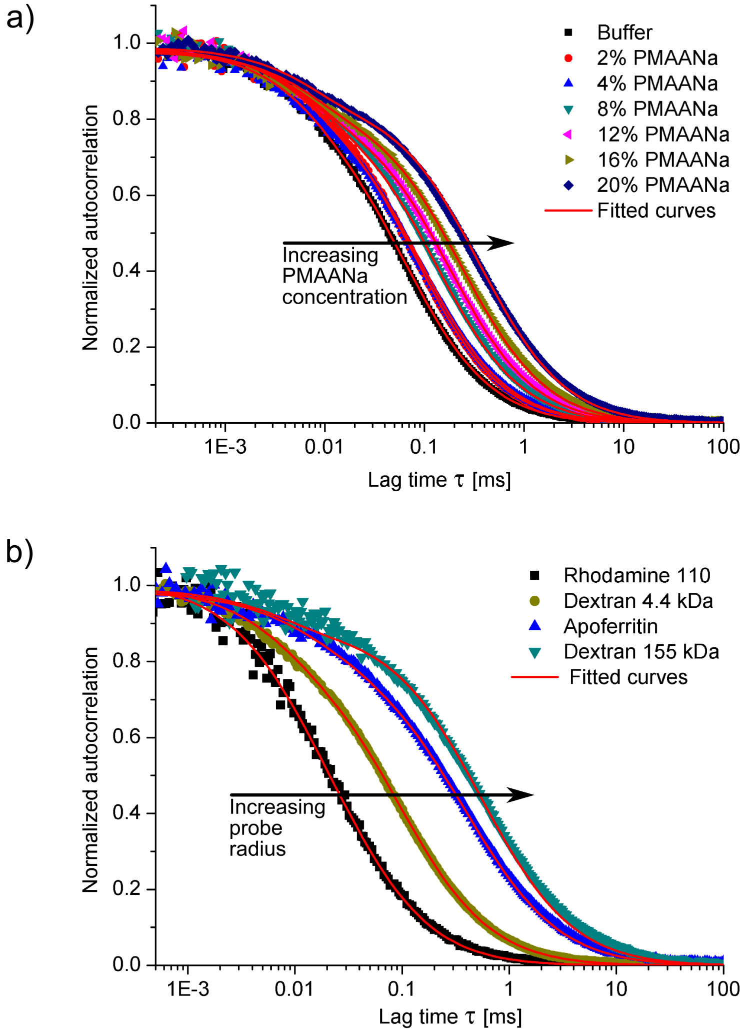

Fig 1. Representative autocorrelation curves form fluorescence correlation spectroscopy experiments. The presented data concern measurements in 19 kDa PMAANa solutions. Each plotted dataset is an average of at least 10 independent measurements. Fitting was performed in Gnuplot software using a standard 3D diffusion model with Gaussian detection profile and including triplet states. Panel a) shows the increase of the diffusion time of 4.4 kDa dextran with the polyelectrolyte concentration; panel b) shows the increase of the diffusion time of the probes with the probe radius for a 4% (w/w) solution of 18 kDa PMAANa.
